# Bronchiolitis Obliterans Organizing Pneumonia as an Initial Presentation of Systemic Lupus Erythematosus: A Rare Case Report and Literature Review

**DOI:** 10.1155/2016/8431741

**Published:** 2016-04-21

**Authors:** Hung-Ping Wang, Chun-Ming Chen, Yih-Yuan Chen, Wei Chen

**Affiliations:** ^1^Division of Allergy, Immunology and Rheumatology, Chiayi Christian Hospital, Chiayi 60002, Taiwan; ^2^Department of Internal Medicine, Ditmanson Medical Foundation Chiayi Christian Hospital, Chiayi 60002, Taiwan; ^3^Division of Pulmonary and Critical Care Medicine, Ditmanson Medical Foundation Chiayi Christian Hospital, Chiayi 60002, Taiwan; ^4^College of Nursing, Dayeh University, Changhua 51591, Taiwan; ^5^Department of Respiratory Therapy, China Medical University, Taichung 40402, Taiwan

## Abstract

The etiology of bronchiolitis obliterans organizing pneumonia (BOOP) remains controversial. While it has been reportedly associated with several connective tissue disorders, there are only rare reports of BOOP associated with systemic lupus erythematosus (SLE). Herein, we report a 56-year-old female patient who presented with dyspnea on exertion, cough, fever, and joint pain of her left wrist and fingers as initial symptoms. Laboratory tests revealed positivity for anti-nuclear antibody, anti-Ro, and anti-double strand DNA antibody. In this case, the patient with SLE had respiratory illness as the initial symptom due to BOOP in the absence of clear etiology. The diagnosis of BOOP was confirmed by thoracic surgery biopsy. Her respiratory symptoms and radiologic findings significantly improved following prednisolone treatment.

## 1. Introduction

Bronchiolitis obliterans organizing pneumonia (BOOP) is characterized histologically by the formation of plugs of fibrous tissue in the alveolar ducts, alveoli, and the distal bronchiole [1]. The etiologies of BOOP were reported, associated with various disorders including infection, inhalation exposure to toxins, drugs, and collagen vascular diseases [2]. Although systemic lupus erythematosus (SLE) has various pulmonary manifestations, initial presentation with BOOP is rare. Notably, patients with BOOP respond well to systemic steroid therapy. Therefore, such patients may heal promptly without further complications. Herein, we present a case of SLE that initially manifested as BOOP. Previously published case reports were collected and distinctive features were compared with the current case.

## 2. Case Presentation

A 56-year-old woman was admitted to our hospital for dyspnea on exertion. She had fever, dry cough, and joint pain of her left wrist and fingers. Chest examination showed an alveolar patch in both lower lung fields. Peripheral white blood cell count on admission was 3.93 × 10^9^/L with 64% neutrophils, 22% lymphocytes, and 13% monocytes. The hematocrit value was 32.7%. The results of urinalysis and C-reactive protein levels were normal. The erythrocyte sediment rate was 95 mm/h.

On admission, physical examination revealed mild pale conjunctiva and bilateral basal crackles over the lung field. Inflammation was observed over the left wrist and secondary metacarpal phalangeal and third proximal interphalangeal joints; the patient complained of pain. There was no excess hair loss, no malar rash, oral ulcer, neck lymphadenopathy, active skin rash, or cyanotic tips. The heart rate was 82 beats/min in regular heart sound. The abdomen was flat and soft without tenderness, and the patient reported normal bowel activity. She had no history of smoking, chemical-agent exposure, alcohol consumption, betel nut chewing, and animal breeding. She also denied any family history of autoimmune disease.

After admission to the ward, intravenous empirical antimicrobial therapy including Augmentin 1200 mg/8 h and azithromycin (Zithromax®) 500 mg/day for 3 continuous days was prescribed for atypical pneumonia. The serological tests for clinical pathogens including* Mycoplasma*,* Legionella,* and* Cryptococcus* were negative. Sputum examination revealed no acid-fast organisms or fungus. After one-week observation, levofloxacin therapy had no effect on symptoms of fever, cough, and dyspnea on exertion. Notably, serological examinations revealed positivity for anti-nuclear antibodies (ANA; titer: 160), anti-Ro (26.7 U/mL), and anti-ds-DNA (56.0 IU/mL).

**Figure 1:**
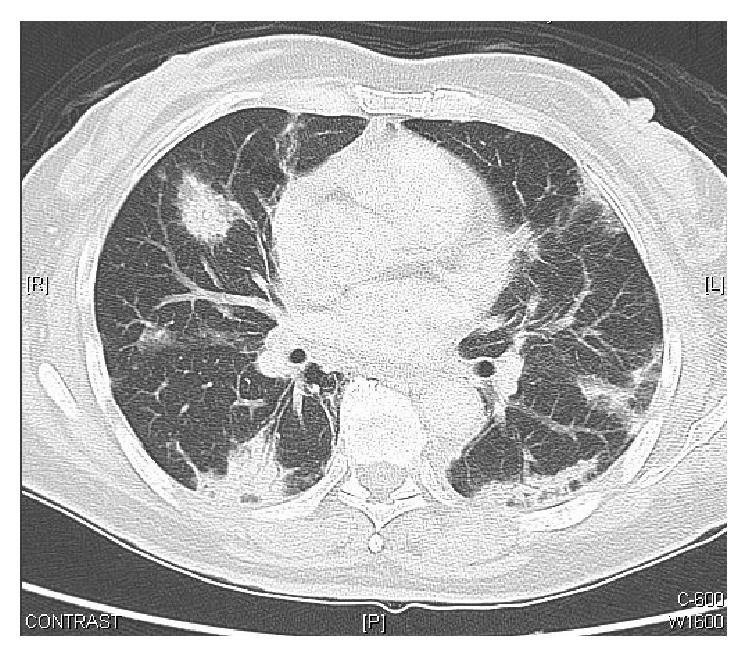
High-resolution computed tomography scans show peribronchial and subpleural bilateral ground-glass opacities with air bronchograms, some with pleural-based triangular shape.

**Figure 2:**
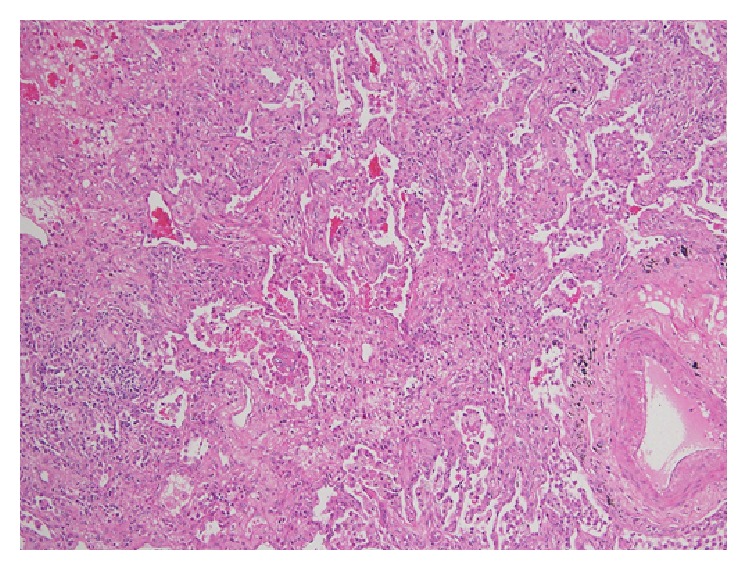
Lung biopsy shows inflammatory debris and foamy histiocytes within small airways, alveolar ducts, and adjacent alveoli (hematoxylin-eosin stain, ×100).

In this case, the patient's symptoms did not improve with the above treatment. Further laboratory studies revealed leukopenia (1.94 × 10^9^/L). A high-resolution computed tomography (HRCT) scan showed peribronchial and subpleural bilateral ground-glass opacities with air bronchograms, some with a pleural-based triangular shape (Figure 1). Although BOOP was the radiologist's first impression, bronchoalveolar cancer was excluded. Video-assisted thoracic surgery biopsy was performed, and the pathology showed a picture of lung-tissue dense lymphohistiocytic infiltration, the airway and alveoli filled with swirls of inflammatory granulation tissue, and formation of fibroblastic plugs (Figure 2). Her biopsy examinations confirmed BOOP (Figure 2). The patient showed continued improvement in her dyspnea on exertion and joint pain of wrist and fingers and white blood cell counts with methylprednisolone, 120 mg daily. The patient was then treated with prednisolone (30 mg/day), hydroxychloroquine (400 mg/day), and azathioprine (50 mg/day) after 1 week of high-dose systemic steroid treatment. The prednisolone dose was tapered at the outpatient department to 5–10 mg/day within 1 month. Three months after treatment, HRCT showed disappearance of all abnormal findings.

## 3. Discussion

BOOP is originally differentiated from organizing pneumonia, which is characterized by formation of granulation tissue in the distal air space, but when it presented with granulation tissue plugs within lumens of small airways, the term bronchiolitis obliterans was added to OP [3]. The precision mechanism of injury leading to the formation of BOOP remains controversial. However, it was reported that injury to the alveolar epithelium plays an important role [1]. Al-Ghanem et al. indicated that the pathogenic mechanism of BOOP is that of an inflammatory lung disease rather than a fibrosing process [3].

BOOP was also revealed in association with several connective tissue disorders, including rheumatoid arthritis, mixed connective tissue disease, and dermatomyositis-polymyositis [1, 3, 4]. In the past decades, BOOP was still reported as a rare complication of SLE. Notably, Epler, Otsuka, and Takada et al. have described several cases of BOOP in patients with SLE [1, 5, 6]. As per the report of Takada et al., patients with SLE are commonly affected by respiratory manifestations including acute pneumonitis, alveolar hemorrhage, diaphragmatic dysfunction, interstitial fibrosis, and pleuritis [6]. Therefore, BOOP in association with SLE must carefully exclude other pulmonary disorders. Among those pulmonary disorders, pleuritis is most common in SLE accounting for 40–60% of all complications [7].

The preferred diagnosis method of BOOP is lung biopsy. However, conventional radiography and CT serve as a guide in determining the biopsy site. Moreover, pathologic examination is needed to exclude infection and other pulmonary manifestations. We noticed that inflammatory response in BOOP seemed different from other pulmonary inflammatory diseases such as asthma, chronic obstructive pulmonary disease (COPD), and granulomatous lung disease [3]. Early differential diagnosis is important because treatment might be ineffective against the wrong type of inflammatory response. In our case, the patient presented with BOOP accompanied by arthritis, leukopenia, and presence of antibodies against ANA, ds-DNA, and Ro. Moreover, serological and sputum tests revealed no evidence of infection. Notably, we also compared clinical symptoms with previous studies. We found that clinical symptoms including joint pain, fever, dyspnea, pancytopenia, and presence of ANA, ds-DNA, Sm, and RNP antibodies were also described by Takada et al. in a patient with SLE who had respiratory illness as the initial symptom due to BOOP. In two cases, the diagnosis of BOOP was then confirmed by thoracic surgery biopsy. Thus, more attention should be given to the BOOP aspect of SLE.

## 4. Conclusion

We described a patient with SLE who manifested respiratory illnesses due to BOOP. In addition, HRCT and pathological examinations can be used for early differential diagnosis of pulmonary disease in patients with SLE.
